# Masitinib Inhibits Hepatitis A Virus Replication

**DOI:** 10.3390/ijms24119708

**Published:** 2023-06-03

**Authors:** Reina Sasaki-Tanaka, Toshikatsu Shibata, Mitsuhiko Moriyama, Hirofumi Kogure, Asuka Hirai-Yuki, Hiroaki Okamoto, Tatsuo Kanda

**Affiliations:** 1 Division of Gastroenterology and Hepatology, Department of Medicine, Nihon University School of Medicine, 30-1 Oyaguchi-kamicho, Itabashi-ku, Tokyo 173-8610, Japan; toshimei@gmail.com (T.S.); moriyama.mitsuhiko@nihon-u.ac.jp (M.M.); kogure.hirofumi@nihon-u.ac.jp (H.K.); 2 Division of Experimental Animal Research, National Institute of Infectious Diseases, Tokyo 208-0011, Japan; ahirai@nih.go.jp; 3 Division of Virology, Department of Infection and Immunity, Jichi Medical University School of Medicine, Shimotsuke, Tochigi 329-0498, Japan; hokamoto@jichi.ac.jp

**Keywords:** drug screening, HAV, HuhT7 cells, masitinib, HAV stable replicon

## Abstract

The hepatitis A virus (HAV) infection causes acute hepatitis. HAV also induces acute liver failure or acute-on-chronic liver failure; however, no potent anti-HAV drugs are currently available in clinical situations. For anti-HAV drug screening, more convenient and useful models that mimic HAV replication are needed. In the present study, we established HuhT7-HAV/Luc cells, which are HuhT7 cells stably expressing the HAV HM175-18f genotype IB subgenomic replicon RNA harboring the firefly luciferase gene. This system was made by using a PiggyBac-based gene transfer system that introduces nonviral transposon DNA into mammalian cells. Then, we investigated whether 1134 US Food and Drug Administration (FDA)-approved drugs exhibited in vitro anti-HAV activity. We further demonstrated that treatment with tyrosine kinase inhibitor masitinib significantly reduced both HAV HM175-18f genotype IB replication and HAV HA11-1299 genotype IIIA replication. Masitinib also significantly inhibited HAV HM175 internal ribosomal entry-site (IRES) activity. In conclusion, HuhT7-HAV/Luc cells are adequate for anti-HAV drug screening, and masitinib may be useful for the treatment of severe HAV infection.

## 1. Introduction

Infection with the hepatitis A virus (HAV), a single-stranded and positive-sense RNA virus, is a major cause of acute viral hepatitis and an important public health concern. HAV infection occurs in approximately 1.4 million cases with 27,731 deaths each year in the world [1]. HAV infection induces acute liver failure (ALF), which occurs at lower incidence rates in countries with routine HAV immunization [2] and develops in less than 1% of patients with acute hepatitis A [3,4]. However, the courses of approximately half of patients with ALF result in liver transplant or death [3,4].

A Japanese nationwide survey of ALF and late onset hepatic failure (LOHF) caused by HAV infection revealed that mortality rates between 2004 and 2015 have increased compared to those between 1998 and 2003, mainly because of an increase in underlying metabolic diseases as a consequence of aging [5]. The mean ages of patients who have metabolic diseases and ALF or LOHF caused by HAV infection between 1998 and 2003 (*n* = 45) and between 2004 and 2015 (*n* = 38) are 48 ± 13 years and 58 ± 11 years (*p* < 0.01), respectively. The rates of patients with metabolic diseases and ALF or LOHF caused by HAV infection between 1998 and 2003 (*n* = 45) and between 2004 and 2015 (*n* = 38) are 22% (10/45) and 61% (23/38) (*p* < 0.01), respectively. Diabetic mellitus was more common among deceased patients than among rescued patients (29% (7/24) vs. 8% (4/52); *p* < 0.05) in patients without liver transplantation [5]. Patient age was significantly and independently associated with the outcome of patients with ALF or LOHF caused by HAV infection [5,6].

In several countries, including Japan, where no universal vaccination programs against HAV infection exist and/or hygienic conditions were improved, the number of people without HAV immunity has increased [7]. Therefore, effective antiviral therapies for acute hepatitis A and ALF associated with HAV infection are needed.

Currently, the PiggyBac transposon system is one of the available transposon-based gene delivery systems, and this was originally derived from genomes of baculoviruses that infect the cabbage looper moth *Trichoplusia ni* [8]. The PiggyBac transposon system has been employed for transfection in various mammalian cells, including hepatocytes [9], and is now widely used for stable gene delivery into a broad range of cells types from different species [10]. Esser-Nobis et al. also reported a stable HAV subgenomic replicon system [11]. As it is easier to obtain the stable cell lines using the PiggyBac transposon system, we used this system to establish the stable HAV subgenomic replicon system in the present study.

Masitinib is a member of the class of benzamides [12]. It is a highly selective oral tyrosine kinase inhibitor, an antineoplastic agent and an antirheumatic drug [12]. Masitinib is a potent and selective tyrosine kinase inhibitor targeting c-kit, a member of the type III receptor protein-tyrosine kinase family. Masitinib is effective for the treatment of cancer, mastocytosis and inflammatory diseases [13]. Masitinib is used in the treatment of mast cell tumors in dogs and is available in Europe and in the USA for veterinarians [12].

Here, we established the stable HAV subgenomic replicon system in human hepatoma HuhT7 cells and performed screening for anti-HAV drugs from 1134 US Food and Drug Administration (FDA)-approved drugs. Subsequently, we found that masitinib could inhibit HAV genotypes IB and IIIA replication. Together, the stable HAV subgenomic replicon system harboring the firefly luciferase gene is a very useful and convenient tool for identifying effective HAV-specific antivirals.

## 2. Results

### 2.1. Construction of the HuhT7-HAV/Luc Cells Stably Expressing Hepatitis A Virus (HAV) HM175-18f Genotype IB Subgenomic Replicon Harboring the Firefly Luciferase Gene

**Figure 1:**
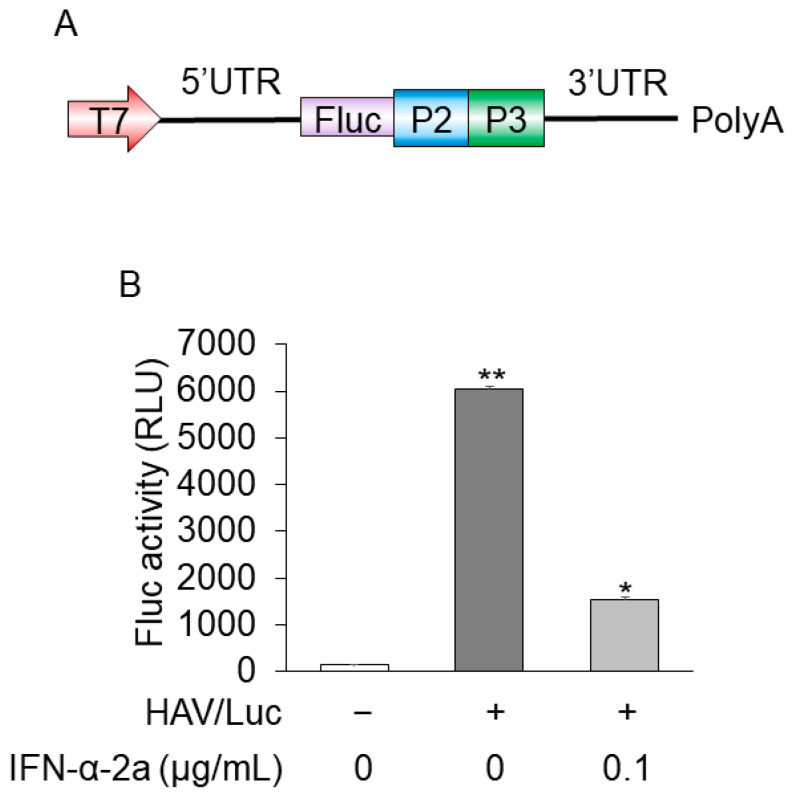
Construction of the HuhT7-HAV/Luc cells. (**A**) Insertion construct of HAV/Luc. (**B**) HuhT7 cells [14] and HuhT7-HAV/Luc cells were seeded at a density of 1 × 10^5^ cells/well in 12-well plates (AGC Techno Glass, Haibaragun, Shizuoka, Japan). HuhT7-HAV/Luc was treated with interferon-α-2a (IFN-α-2a; Sigma-Aldrich, Saint Louis, MO, USA) at 0 or 0.1 μg/mL. After 48 h of incubation, firefly luciferase (Fluc) activity was measured [15,17]. Data are expressed as the means and standard deviations of duplicate determinations from two independent experiments. Statistical significance was determined using a two-tailed Student’s *t* test. * *p* < 0.05, ** *p* < 0.01 (versus untreated control). T7, T7 promoter; P2, HAV P2; P3, HAV P3; 5′UTR, 5′ untranslated region; 3′UTR, 3′ untranslated region; polyA, polyA tail.

For anti-HAV drug screening, to establish more convenient and useful models that mimic HAV replication, we made HuhT7 cells stably express HAV/Luc. The insertion construct of HAV/Luc is shown in Figure 1A. The downstream part of the HAV 5′ untranslated region presents an internal ribosomal entry site (IRES) with a pyrimidine-rich tract that allows RNA translation by a cap-independent mechanism.

Firefly luciferase activities and HAV RNA levels were determined using reporter assays and real-time RT PCR, respectively [14,15]. HuhT7-HAV/Luc cells expressed ~6000 relative luciferase activity units (RLU)/1.5 × 10^5^ cells, and HuhT7-HAV/Luc cells treated with 0.1 μg/mL interferon-α-2a expressed ~1000 RLU/1.5 × 10^5^ cells. Interferon-α-2a inhibited luciferase activity, which was found at a fourth of those in untreated cells, as previously reported [16]. Firefly luciferase activity of parent HuhT7 cells was negligibly small (Figure 1B). Threshold cycle (Ct) numbers of HAV RNA in HuhT7-HAV/Luc and control HuhT7 cells were 23.9 and over 40, respectively, via real-time RT-PCR. Thus, HuhT7-HAV/Luc cells stably expressing the HAV HM175-18f genotype IB subgenomic replicon RNA harboring the firefly luciferase gene were established for drug screening.

### 2.2. Screening for 1134 Drugs Derived from FDA-Approved Drug Library for Anti-Hepatitis A Virus (HAV) Drugs in HuhT7-HAV/Luc Cells

**Figure 2:**
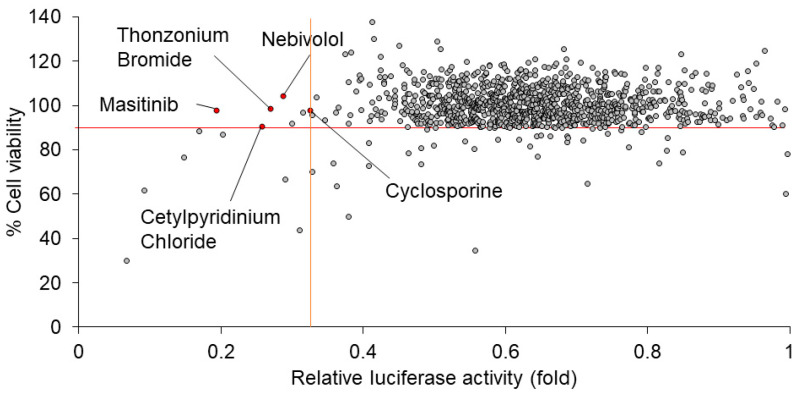
Masitinib is a candidate drug for inhibition of the hepatitis A virus (HAV) HM175-18f genotype IB subgenomic replicon. Analyses of 1134 drugs screened by luciferase assays and dimethylthiazol carboxymethoxyphenyl sulfophenyl tetrazolium (MTS) assays [15,17]. The results of the drug screening are shown. All drugs were plotted on a scattergram in which the *Y*-axis and *X*-axis indicate the % cell viability of HuhT7-HAV/Luc cells and the relative luciferase activity (fold) of HAV/Luc, respectively. Masitinib, cetylpyridinium chloride, nebivolol, cyclosporine and thonzonium bromide are indicated as red circles. The red horizontal line indicates 90% cell viability. The orange vertical line indicates 0.33-fold relative luciferase activity.

We performed screening for 1134 drugs derived from the FDA-approved Drug Library using HuhT7-HAV/Luc cells. To screen the drugs which inhibit firefly luciferase activity derived from HAV/Luc cells, the drug screening assay was applied following the optimization of multiple parameters, including firefly luciferase activity and cell viability, using HuhT7-HAV/Luc cells. To select the drugs with higher efficacy and lower cytotoxicity, relative luciferase activity of HAV/Luc < 0.33-fold and cell viability > 90% were initially chosen as criteria for the further confirmation studies of HAV infection (Figure 2). From among the 1134 drugs derived from the FDA-approved Drug Library, we selected five candidates as anti-HAV drugs: masitinib, cetylpyridinium chloride, nebivolol, cyclosporine and thonzonium bromide. Sunitinib malate and alexidine hydrochloride also met the criteria above; however, these drugs were excluded from the present study due to the following reasons: sunitinib has been used for the treatment for liver cancer [18], and alexidine hydrochloride was not available.

### 2.3. Masitinib Significantly Inhibits Hepatitis A Virus (HAV) HA11-1299 Genotype IIIA Replication

**Figure 3:**
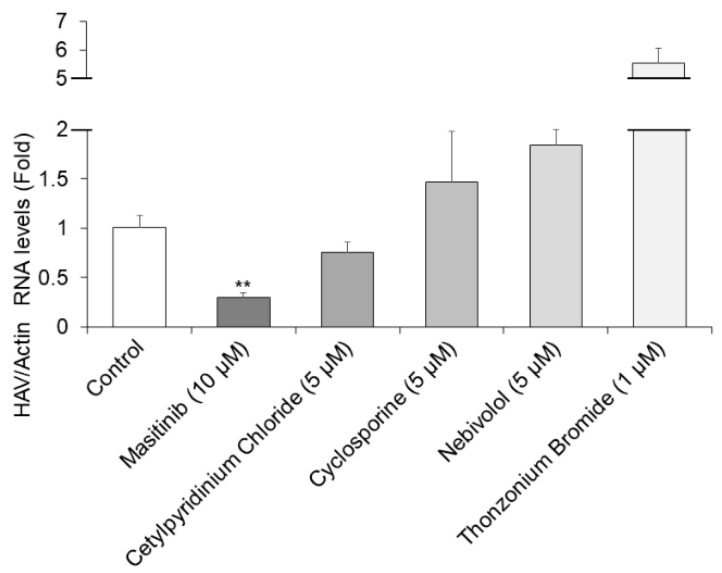
Masitinib inhibits hepatitis A virus (HAV) HA11-1299 genotype IIIA infection. Huh7 cells infected with the HAV HA-11-1299 genotype IIIA strain were treated with 10 μM masitinib; 5 µM cetylpyridinium chloride, nebivolol and cyclosporine; and 1 µM thonzonium bromide for 72 h. HAV RNA levels were examined using real-time RT-PCR [15,17,20]. Actin mRNA was used as an internal control. HAV RNA levels were significantly inhibited in masitinib-treated Huh7 cells. Data are expressed as the means and standard deviations of triplicate determinations from three independent experiments. Statistical significance was determined using a two-tailed Student’s *t* test. ** *p* < 0.01.

Further analysis for the selected five compounds was performed following the optimization of multiple parameters, including HAV RNA inhibition and cell viability using Huh7 cells [19]. As the five candidates were selected as anti-HAV drugs at 10 µM each in HuhT7-HAV/Luc cells, we next used 10 µM or lower of each drug to inhibit HAV HA11-1299 genotype IIIA replication in Huh7 cells. The concentration of these drugs indicated in Figure 3 had no cytotoxicity on Huh7 cells. Out of the initial five hits, masitinib was re-confirmed to have no cytotoxic activity at 10 µM or lower in these culture conditions. Cetylpyridinium chloride, nebivolol and cyclosporine showed no cytotoxic activity at concentrations of 5 µM or lower, and thonzonium bromide showed no cytotoxic activity at concentrations of 1 µM or lower.

RT-qPCR was performed for the detection of HAV RNA [20]. Masitinib treatment at a concentration of 10 µM resulted in a 71% reduction in HAV HA11-1299 genotype IIIA replication in Huh7 cells (Figure 3). Thus, 10 µM masitinib significantly inhibited HAV HA11-1299 genotype IIIA replication, compared to the untreated control. Unfortunately, the other drugs had no significant inhibitory effects on HAV HA11-1299 genotype IIIA replication. We have previously used 72 h drug treatments for detecting the effects of drugs on HAV replication using RT-qPCR [17,21]. In this study, we used this system for detecting the effects on HAV replication, although the half-life (t 1/2) of masitinib is ~5 h.

### 2.4. Masitinib Significantly Inhibits Hepatitis A Virus (HAV) HM175-18f Genotype IB Replication

**Figure 4:**
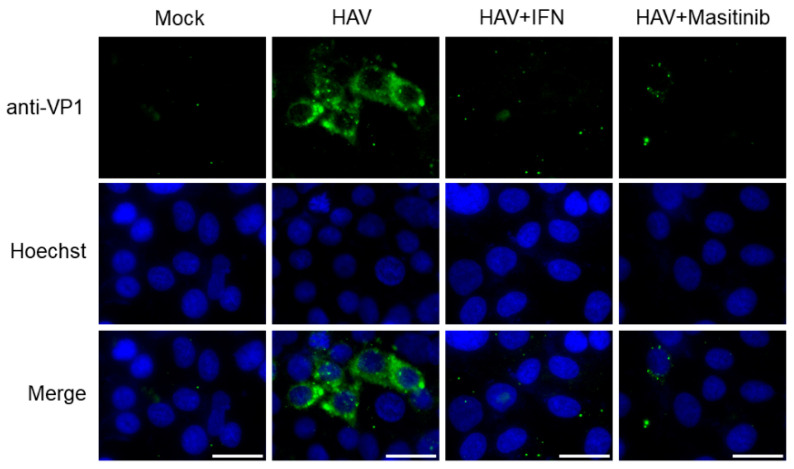
Immunofluorescence analysis of hepatitis A virus (HAV) HM175-18f genotype IB-infected Huh7 cells. Antibodies against HAV VP1 are shown in green, and nuclei stained with Hoechst are shown in blue [17,21]. Positive immunofluorescence staining observed for HAV VP1 in HAV-infected cells but not in the uninfected cells (Mock) [21]. HAV HM175-18f genotype IB-infected Huh7 cells treated with 10 µM masitinib reduced HAV VP1 staining. HAV HM175-18f genotype IB-infected Huh7 cells treated with 0.1 μg/mL interferon-α-2a (IFN) stained with HAV VP1 as the control. Scale bar: 25 μm.

To examine the effects of masitinib on HAV HM175-18f genotype IB replication in Huh7 cells, we further determined the HAV VP1 expression with immunofluorescence staining (Figure 4) [17,21]. We demonstrated that HAV HM175-18f genotype IB-infected Huh7 cells expressed HAV VP1, which were observed in the cytoplasm of infected cells (Figure 4). Meanwhile, HAV VP1 expression decreased in HAV HM175-18f genotype IB-infected Huh7 cells treated with masitinib or interferon-α-2a (positive control) when compared to those without (Figure 4). Thus, masitinib significantly reduced both HAV HM175-18f genotype IB replication and HAV HA11-1299 genotype IIIA replication.

### 2.5. Masitinib Significantly Inhibits Hepatitis A Virus (HAV) HA11-1299 Genotype IIIA Replication in a Dose-Dependent Manner

HAV RNA was significantly reduced in HAV HA11-1299 genotype IIIA-infected Huh7 cells treated with masitinib. Masitinib treatment at concentrations of 5 μM and 10 μM resulted in 32% and 60% reductions in HAV RNA levels, respectively. Masitinib inhibited HAV replication in a dose-dependent manner, with an estimated half maximal inhibitory concentration (IC_50_) of 7.81 μM [22], although the HAV/Actin RNA level in the 0.5 μM group was not significantly different from those in the 0.1 μM or 1 μM groups.

### 2.6. Masitinib Significantly Inhibits the Activity of the Hepatitis A Virus (HAV) HM175 Genotype IB Internal Ribosomal Entry Site (IRES)-Mediated Translation in a Dose-Dependent Manner

**Figure 5:**
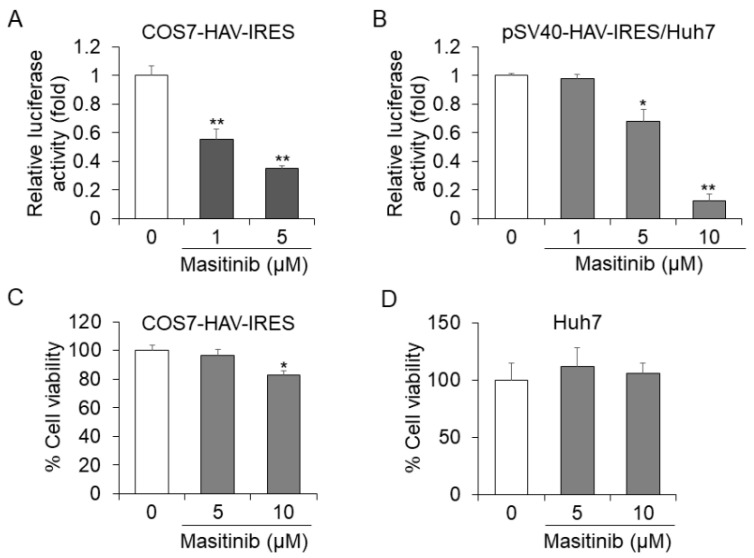
Masitinib inhibits hepatitis A virus (HAV) internal ribosomal entry site (IRES)-mediated translation. (**A**) COS7-HAV-IRES cells were seeded at a density of 1 × 10^5^ cells/well in 12-well plates (AGC Techno Glass). COS7-HAV-IRES was treated with masitinib at 0, 1 and 5 μM. After 48 h of incubation, a luciferase assay was performed. (**B**) Huh7 cells were seeded 24 h prior to transfection at a density of 1 × 10^5^ cells/well in 12-well plates. Cells were transiently transfected with 0.2 μg of pSV40-HAV-IRES using Effectene Transfection Reagent (Qiagen, Chuo-ku, Tokyo, Japan ). After 24 h of transfection, the cells were treated with masitinib at 0, 1, 5 and 10 μM. After 72 h of transfection, luciferase activities were determined. The cytotoxicity of masitinib on COS7-HAV-IRES and Huh7 cells was determined (**C**,**D**). COS7-HAV-IRES and Huh7 cells were treated with masitinib at 0, 5 and 10 μM for 48 h. Cell viability was measured via dimethylthiazol carboxymethoxyphenyl sulfophenyl tetrazolium (MTS) assays. Data are expressed as the means and standard deviations of triplicate determinations from three independent experiments. We compared statistical significance using the Student’s *t* test in two independent groups: a sample group and a control group. * *p* < 0.05, ** *p* < 0.01.

In HuhT7-HAV/Luc cells, the firefly luciferase gene is located downstream of HAV IRES. It is possible that the drug screening assay using HuhT7-HAV/Luc cells might also be designed to identify drugs that inhibit HAV IRES-mediated translation. To examine the intracellular mechanisms modulated by masitinib in HAV-infected cells, we focused on the HAV IRES and examined whether tyrosine kinase inhibitor masitinib regulates the HAV translation through HAV IRES inhibition. We investigated the effect of masitinib on HAV IRES activity using a different construct by luciferase assay. Masitinib treatment at a concentration of 5 µM resulted in 66% and 33% reductions of HAV IRES activity in COS7-HAV-IRES cells and Huh7 cells transfected with pSV40-HAV-IRES, respectively, compared with each control (Figure 5A,B) [16,21,23,24]. Using a concentration of 10 µM, masitinib resulted in an 88% reduction in Huh7 cells transfected with pSV40-HAV-IRES (Figure 5B). Masitinib inhibited HAV IRES activity in COS7-HAV-IRES and Huh7 cells in a dose-dependent manner (Figure 5A,B). As shown in Figure 5C,D, masitinib did not impact the cell viabilities of COS7-HAV-IRES cells or Huh7 cells, both at concentrations of 5 μM. Thus, we confirmed that masitinib inhibits HAV IRES-mediated translation using two different assays.

## 3. Discussion

In the present study, we successfully developed HuhT7-HAV/Luc cells to stably express the HAV HM175-18f genotype IB subgenomic replicon RNA harboring the firefly luciferase gene. Subsequently, we investigated whether 1134 FDA-approved drugs exhibit in vitro anti-HAV activity using this model. HAV genotypes I and III are major HAV genotypes in Japan and other countries [25,26,27]. Masitinib significantly reduced both HAV HM175-18f genotype IB replication and HAV HA11-1299 genotype IIIA replication. Finally, we identified the efficacy of masitinib with respect to its inhibition of replication of multiple HAV genotypes through the inhibition of HAV IRES activity.

We and others have performed screening of antiviral compounds for inhibitory effects on HAV replication using a combination of the HAV cell culture system with HAV antigen ELISA [28,29], or RT-PCR [30]; the transient transfection system of HAV subgenomic replicon harboring the firefly luciferase gene [15,21]; COS7-HAV-IRES cells [21]; and an in silico approach [31].

In the present study, we used a PiggyBac-based gene transfer system that introduces nonviral transposon DNA into mammalian cells to establish HuhT7-HAV/Luc cells because the HAV/Luc is a relatively long DNA sequence (~7000 bp). An HuhT7-HAV/Luc cell line, in which higher luciferase activities were observed, was established and is an appropriate tool to shorten the process for drug repositioning (Figure 1B).

Masitinib is a selective oral tyrosine kinase inhibitor, exerting experimental neuroprotection via targeting microglia, macrophages and mast cell activities and their immunomodulatory properties, in both central and peripheral nervous systems [32,33,34]. Masitinib has a promising preclinical activity in amyotrophic lateral sclerosis (ALS) rat models [32]. The antiviral effects of several tyrosine kinase inhibitors were reported. The tyrosine kinase inhibitor dasatinib blocks in vitro HIV-1 production by primary CD4+ T cells in HIV-1-infected patients [35]. Dasatinib inhibits HIV-1 replication through the interference of SAM- and HD domain-containing deoxynucleoside triphosphate triphosphohydrolase 1 (SAMHD1) phosphorylation in CD4+ T cells [36]. The oral multiple kinase inhibitor sorafenib attenuates HCV replication by inhibiting Raf-1 kinase, which is associated with the hepatitis C virus (HCV) NS5A, and regulates HCV replication [37]. Sorafenib inhibits multiple steps of the HCV infectious cycle in vitro [37,38,39].

Tyrosine kinase is known to regulate IRES-mediated translation [40], and we have previously shown that the tyrosine kinase inhibitor AG490 regulates HAV IRES-mediated translation [41]. In the present study, we focused on the HAV IRES activity in Huh7 cells treated with masitinib, and we revealed that masitinib inhibits HAV IRES-mediated translation (Figure 5A,B).

We have recently reported that nicotinamide inhibits c-Jun expression and HAV replication through the inhibition of HAV IRES-mediated translation [21]. The association between masitinib and c-Jun expression was reported [42,43]. Concerning the mechanistic details of masitinib for the inhibition of HAV IRES-mediated translation, we examined the effects of masitinib on c-Jun expression by Western blot analysis. However, we did not observe any remarkable change between HAV-infected Huh7 cells treated with or without masitinib.

Masitinib targets the proto-oncogene c-kit, which is predominantly expressed in mast cells [44]. The c-kit ligand stem cell factor (SCF) induces several signal transduction pathways through receptor phosphorylation, including the mammalian target of rapamycin (mTOR) pathway [45]. We have previously reported that HAV replication was inhibited through mTOR inhibition [17]; however, there was no remarkable change in phosphorylated and total mTOR expression levels between HAV-infected Huh7 cells treated with or without masitinib by Western blot analysis.

Recently, masitinib has been reported as a severe acute respiratory syndrome coronavirus 2 (SARS-CoV-2) inhibitor which blocks the proteolytic activity of SARS-CoV-2 main protease (M^pro^/3CLpro) [46,47]. Molecular dynamics simulations of the masitinib-M^pro^ complex crystal structure and X-ray crystallography show that masitinib acts as a competitive inhibitor of M^pro^/3Clpro [46,47]. In the present study, masitinib works as an antiviral against HAV in vitro. Masitinib may work as a protease inhibitor to suppress HAV replicon replication and HAV HA11-1299 genotype IIIA replication. Molecular docking studies may reveal the possible efficacy of masitinib as an HAV protease inhibitor on HAV replication [31].

It has been reported that the infection of apathogenic double-stranded RNA (dsRNA) vaccine virus candidate, the infectious bursal disease virus (IBDV) that activates the interferon stimulated genes, is the natural antiviral defense system of host cells [48]. The coinfection of apathogenic dsRNA virus and other viruses are also referred to viral superinfection therapy (SIT). SIT has already been demonstrated to be safe and effective against five different families of viruses: HAV, hepatitis B virus, HCV, SARS-CoV-2 and herpes zoster virus [48]. Thus, new intentional viral coinfection therapy may also be a promising anti-HAV approach [48]. Host-targeting agents as well as direct-acting antiviral agents could be useful for the control of HAV infection [49,50].

In the present study, we focused on 1134 FDA-approved drugs because we investigated potentially effective drugs by drug repositioning [21,22]. The choice of existing drugs has the potential to shorten the process. As masitinib has been approved by the FDA, several required clinical trial steps could be skipped, and the time and costs for its appearance into markets could also be reduced [51]. One of the limitations of our study is that we did not examine whether masitinib could inhibit HAV replication in vivo as there are no inexpensive animal models for the drug screening of HAV. Further study will be needed in this regard.

## 4. Materials and Methods

### 4.1. Cells and Reagents

The human hepatoma cell lines Huh7 and HuhT7, which are stably transformed derivatives of Huh7 expressing T7 RNA polymerase in the cytoplasm, were used [15,19]. Huh7 and its derived cell lines could support HAV replication well [52]. Huh7 and HuhT7 cells were kindly provided by Prof. Bartenschlager and Prof. Gauss-Müller, respectively [14,19]. The African green monkey kidney cell line COS7-HAV-IRES, which stably expresses the HAV IRES followed by firefly luciferase, was also used [15,23]. The cells were maintained in a Roswell Park Memorial Institute medium (RPMI; Sigma-Aldrich) containing 10% heat-inactivated fetal bovine serum (FBS; Sigma-Aldrich), 100 units/mL penicillin and 100 μg/mL streptomycin (Sigma-Aldrich) with 5% CO_2_ at 37 °C.

The HAV HA11-1299 genotype IIIA strain and HAV HM175-18f genotype IB strain, which were kindly provided by Prof. Lemon, were used for HAV infection in the present study [17]. Replication-competent HAV HM175 genotype IB subgenomic replicon (HAV subgenomic replicon) pT7-18f-LUC, containing an open reading frame of firefly luciferase flanked by the first four amino acids of the HAV polyprotein and by 12 C-terminal amino acids of VP1 (HAV HM175-18f genotype IB strain), was kindly provided from Prof. Gauss-Müller [14]. The structure of pT7-18f-LUC has been documented previously [14,16]. The plasmid pSV40-HAV-IRES was previously described [16,24].

The FDA-approved Drug Library, including 1134 drugs, was kindly provided by the Center for Supporting Drug Discovery and Life Science Research, Graduate School of Pharmaceutical Sciences, Osaka University, Suita, Osaka, Japan. Masitinib, cetylpyridinium chloride, nebivolol and cyclosporine were purchased from Selleck Biotek (Taito-ku, Tokyo, Japan). Thonzonium bromide was purchased from Toronto Research Chemicals Inc. (Toronto, ON, Canada).

### 4.2. Establishment of HuhT7-HAV/Luc Cells Stably Expressing HAV Subgenomic Replicon RNA Harboring the Firefly Luciferase Gene

HAV/Luc was amplified by polymerase chain reaction (PCR) from pT7-18f-LUC with KOD-plus-Neo polymerase (Toyobo, Kita-ku, Osaka, Japan). Primer sets are listed in Table 1. The PCR was performed as follows: 94 °C for 2 min, followed by 40 cycles of 98 °C for 10 s, 55 °C for 5 s and 68 °C for 4 min, and followed by 68 °C for 3 min, on a GeneAmp PCR system 9700 (Perkin Elmer, Norwalk, CT, USA).

The PiggyBac Transposon is a mobile genetic element that efficiently transposes between vectors and chromosomes. Powerful activity of the PiggyBac Transposon system enables genes of interest between the two transposon-specific inverted terminal repeat sequences (ITRs) located on both ends of the PiggyBac Transposon vector to be easily mobilized into target genomes [53]. To establish the PiggyBac Transposon vector encoding HAV/Luc, the PCR fragments were cloned into the EcoR1 site of nonviral PiggyBac Transposon vector (PB501B-1; System Biosciences, Palo Alto, CA, USA) using the In-Fusion cloning kit (Takara Bio, Kusatsu, Shiga, Japan). To establish cells that stably express HAV/Luc, HuhT7 cells were co-transfected with nonviral PiggyBac Transposon vector encoding HAV/Luc and PiggyBac Transposase vector (PB210PA-1; System Biosciences) using Effectene transfection reagents (Qiagen). After 96 h of transfection, cells were treated with 5 μg/mL puromycin (Thermo Fisher Scientific, Koto-ku, Tokyo, Japan) for the selection of HuhT7-HAV/Luc cells. After 2 weeks, to avoid monoclonal selection, all cells were collected for further analysis.

### 4.3. Drug Screening in HuhT7-HAV/Luc Cells Stably Expressing HAV HM175 Genotype IB Subgenomic Replicon RNA Harboring the Firefly Luciferase Gene

The drug screening was performed in a 96-well plate format MS-8096W (Sumitomo Bakelite, Shinagawa, Tokyo, Japan). HuhT7-HAV/Luc cells were plated at a density of 2 × 10^4^ to 2.5 × 10^4^ cells/well. After 24 h, cells were treated with 10 µM of each of the 1134 drugs or DMSO alone. In the screening assay, DMSO was used as the control. After 24 h, luciferase activities were determined as HAV subgenomic replicon replication using luciferase reporter assays, and cell viabilities were determined with dimethylthiazol carboxymethoxyphenyl sulfophenyl tetrazolium (MTS) assays (Promega, Madison, WI, USA), as described previously [22].

### 4.4. Luciferase Reporter Assays

The cells were harvested using reporter lysis buffer (Promega), and luciferase activities were determined with a PicaGene luminescence kit (Toyo Ink, Chuo-ku, Tokyo, Japan), and luciferase activities were determined using a luminometer (AB-2200-R, ATTO, Taito-ku, Tokyo, Japan). The luciferase activity fraction (percent) was calculated as follows: [(test compound − blank)/(DMSO − blank)] × 100. Luciferase activities presented are averages from three independent experiments, as previously described [21].

### 4.5. Cell Viability Assays

For the evaluation of cell viability, MTS assays were performed using the CellTiter 96 Aqueous One-Solution cell proliferation assay (Promega). Enzyme activity was measured with a Bio-Rad iMark microplate reader (Bio-Rad, Hercules, CA, USA) at the 490 nm wavelength [15,17]. 

### 4.6. Examination of Anti-HAV Activities of Selected Drugs in HAV HA11-1299 Genotype IIIA-Infected Huh7 Cells

Huh7 cells were seeded 24 h prior to infection at a density of 3 × 10^5^ cells/well in 6-well plates (AGC Techno Glass). The cells were washed twice with phosphate-buffered saline (PBS) (Fujifilm Wako Pure Chemical Corporation, Chuo-ku, Osaka, Japan) and infected with the HAV HA11-1299 genotype IIIA strain at a multiplicity of infection (MOI) of 0.1 in serum-free RPMI. Then, 1 μM thonzonium bromide; 5 μM cetylpyridinium chloride, nebivolol and cyclosporine; 10 μM masitinib; and serum-free RPMI (as a control) were added to HAV-infected Huh7 cells. After 24 h of incubation, the cells were washed once with PBS, followed by the addition of 1 mL of RPMI containing 5% FBS, with or without 1 μM thonzonium bromide; 5 μM cetylpyridinium chloride, nebivolol and cyclosporine; and 10 μM masitinib. After 72 h of infection, the HAV RNA levels in the inoculated cells were determined using real-time reverse transcription-polymerase chain reaction (RT-PCR), according to the following instructions.

### 4.7. RNA Extraction and Quantification of HAV RNA and Actin mRNA

Total cellular RNA was extracted from harvested cells using the RNeasy Mini Kit (Qiagen, Chuo-ku, Tokyo, Japan) according to the manufacturer’s instructions. cDNA was synthesized using the PrimeScript RT reagent (Perfect Real Time; Takara Bio). Reverse transcription was performed at 37 °C for 15 min, followed by 85 °C for 5 s. Real-time PCR was performed using Power SYBR Green Master Mix (Thermo Fisher Scientific) with a QuantStudio 3 real-time PCR system (Applied Biosystems, Chuo-ku, Tokyo, Japan). The primer sets for the quantification of HAV RNA and actin mRNA are listed in Table 1 [20]. The PCR reaction was performed as follows: 95 °C for 10 min, followed by 40 cycles of 95 °C for 15 s and 60 °C for 1 min. The actin housekeeping gene was used for normalization, and data were analyzed using the comparative threshold cycle method. Relative quantification of gene expression using the ΔΔCt method correlated with absolute gene quantification obtained by standard curve. Each real-time PCR assay was performed in duplicate.

### 4.8. Calculation of the Half Maximal Inhibitory Concentration (IC_50_) 

The concentrations of masitinib that produce 50% of a maximal inhibition of HAV are IC_50_, which are obtained from the following equation: IC_50_ = 10^[LOG(A/B) × (50 − C)/(D − C) + LOG(B)], as previously described [22]. Variables indicate a higher concentration of two values that sandwich IC_50_ (A), a lower concentration of two values that sandwich IC_50_ (B), HAV RNA levels (%) at B (C), and HAV RNA levels (%) at A (D).

### 4.9. Immunofluorescence Staining

Huh7 cells were seeded 24 h prior to infection at a density of 5 × 10^4^ cells/well on cover slips in a 24-well plate (AGC Techno Glass). The cells were washed twice with PBS (Fujifilm Wako) and infected with the HAV HM175-18f genotype IB strain at a multiplicity of infection (MOI) of 0.1 in serum-free RPMI. Then, 10 μM masitinib and 0.1 μg/mL interferon-α-2a (Sigma-Aldrich) were added in HAV-infected Huh7 cells. After 24 h of incubation, the cells were washed once with PBS, followed by the addition of 500 µL of RPMI containing 5% FBS, with 10 μM masitinib and 0.1 μg/mL of interferon-α-2a. After 72 h of infection, cells were fixed with ice-cold methanol for 5 min at −20 °C. After the removal of methanol, cells were dried for 3 min, then washed with PBS twice. Later, the cells were blocked using a blocking solution (3% BSA in PBS) for 30 min at room temperature, and the cells were incubated for 1 h with a primary antibody against HAV VP1 (anti-HAV VP1 Antibody (aa7-143), 1:50, LS-C137674, LifeSpan BioSciences, Seattle, WA, USA), followed by a secondary antibody Alexa Fluor 488 F(ab’)2 fragment of goat anti-mouse IgG (1:500, A-11017, Thermo Fisher Scientific). Nuclei were stained with 5 μg/mL Hoechst 33342 (Sigma-Aldrich) in PBS for 10 min, and the cover slip was mounted using an anti-fluorescent quencher (SlowFad Gold Antifade Mountant, Thermo Fisher Scientific). Immunofluorescence images were captured under a Keyence BZ-X710 fluorescence microscope (Takatsuki, Osaka, Japan) using 40× objective magnification.

### 4.10. Statistical Analysis

All assays were performed in triplicate. Data are expressed as the mean ± standard deviations (SD). The statistical analysis was conducted using the Microsoft Excel program for Windows 2010 (Minato-ku, Tokyo, Japan). Statistical significance was determined using a two-tailed Student’s *t* test. *p* < 0.05 was considered a statistically significant difference between the two groups.

## 5. Conclusions

HuhT7-HAV/Luc cells stably expressing HAV HM175 genotype IB subgenomic replicon RNA harboring the firefly luciferase gene were established and were useful for anti-HAV drug screening. We demonstrated that masitinib inhibited HAV subgenomic and genomic replication, inhibiting HAV IRES-mediated translation.

## 6. Patents

A patent (Japanese patent application No. 2023-014730; filing date 2 February 2023) based on this research and experiments was applied for (T.K. and R.S.-T.).

## Figures and Tables

**Table 1:** Primers used in the present study.

Targets	Directions	Sequences of Primers
***For making inserts HAV/Luc of PiggyBac Transposon***		
pT7-18f-LUC	Sense	5′-TAGAGCTAGCGAATTTAATACGACTCACTATAGGG-3′
pT7-18f-LUC	Antisense	5′-ATTTAAATTCGAATTGTCAGGTGGCACTTTTCG-3′
***For real-time RT-PCR***		
HAV	Sense	5′-AGGCTACGGGTGAAACCTCTTAG-3′
HAV	Antisense	5′-GCCGCTGTTACCCTATCCAA-3′
Actin	Sense	5′-CAGCCATGTACGTTGCTATCCAGG-3′
Actin	Antisense	5′-AGGTCCAGACGCAGGATGGCATG-3′

HAV, hepatitis A virus; RT-PCR, reverse transcription-polymerase chain reaction.

## Data Availability

The data underlying this article are available in this article.
